# Knowledge, Attitude, and Preventive Practices among Prison Inmates in Ogbomoso Prison at Oyo State, South West Nigeria

**DOI:** 10.1155/2014/364375

**Published:** 2014-09-25

**Authors:** Abdulsalam Saliu, Babatunde Akintunde

**Affiliations:** Department of Community Medicine, Ladoke Akintola University of Technology Teaching Hospital, Ogbomoso 201, Oyo State, Nigeria

## Abstract

Prisoners are at special risk for infection with human immunodeficiency virus (HIV) because of overcrowded prisons, unprotected sex and sexual assault, occurrence of sexual practices that are risky to health, unsafe injecting practices, and inadequate HIV prevention, care, and support services. This study aimed to describe the knowledge, attitude, and preventive practices towards HIV/AIDS by male inmates in Ogbomoso Prison at Oyo State, South West Nigeria. This was a cross-sectional study. A simple random sampling method was employed to select 167 male participants and data were collected using pretested structured interviewer-administered questionnaire. The data were collated and analyzed using the Statistical Package for Social Sciences version 17. Fifty (29.9%) were in the age group 20–24 years with mean age of 30.99 ± 11.41. About half (50.3%) had been married before incarceration. Family and friends (30%), health care workers (25%), prison staff (20%), and mass media (25%) were the commonest sources of information on HIV/AIDS. Knowledge about HIV was found to be high (94.6%). About 68.9% believed that people with the disease should be avoided. The knowledge about HIV/AIDS among inmates was high, but misconceptions about HIV/AIDS are still rife among the prisoners and educational programs would be required to correct this.

## 1. Introduction

Globally, many studies on human immunodeficiency virus/acquired immunodeficiency disease syndrome (HIV/AIDS) have been undertaken by various government and nongovernmental organizations among the general public. There are certain high risk groups in well-defined but restricted settings who are usually left out from the interventions they deserve especially in developing countries. Inmates of prisons are example of this left out population [1]. Prisoners worldwide have a significantly higher prevalence of HIV than in the community [2, 3]. Prisoners are at a special risk for HIV infection because of overcrowded prisons, unprotected sex and sexual assault, occurrence of sexual practices that are risky to health, unsafe injecting practices, and inadequate HIV prevention, care, and support services [4].

Generally in Africa, existing data on HIV/AIDS in prison are not recent or accurate enough to provide a real picture of the current situation [5]. Of particular importance is that documentation of research studies on HIV/AIDS among prison inmates in Nigeria is very scanty and limited [5]. However, there is growing concern over the HIV status of inmates in Nigeria prisons as a report showed that there is an increase in the number of prisoners who are affected with the disease [6]. One of the previous studies found out that HIV is not a silent issue to Nigeria prison inmates which may be a reflection of the generally high level of knowledge of HIV/AIDS among the general population [7]. Other studies equally showed a very high awareness of HIV/AIDS among prison inmates in Nigeria [1–3]. However, despite the high knowledge, misconceptions of various degrees concerning HIV/AIDS were documented in Nigeria and also elsewhere in Africa [7–9].

The common high risk behaviors in the prison environment include rampart use of drugs, practice of tattooing and toothbrush sharing, prison marriages, unprotected violence, rape, sex bartering, sexual assault, and sex among inmates (mostly anal and between males) [10]. Homosexual activity which is culturally, religiously, and politically unacceptable by most societies is widely spread behind the wall and Nigerian prisoners are not an exception [2]. This is because prisons, being unisexual institutions, create an ideal environment for various sexual activities between men [11]. Some inmates are lured by other inmates to have conceptual anal intercourse in exchange for food and toiletries probably due to lack of basic sanitary materials and adequate nutrition in prison [2]. The majority of inmates who engaged in homosexual activities in the prisons are actually circumstantial homosexuals who would not have become involved in the practice if they were not confined [12–14]. A previous study reported that very few of the inmates knew that HIV/AIDS could be contacted through homosexual intercourse [9].

These sexual encounters are fraught with the risk of contracting HIV because of the frequent tearing of sensitive anal membranes. Prisoners are most at risk population not only for HIV and other sexually transmitted infections (STIs) but also for tuberculosis (TB) due to overcrowding, lack of ventilation, and poor prevention practices. TB is the most opportunistic infection among people living with HIV in Africa resulting in high mortality rates among prisoners with HIV/AIDS. Despite the necessity of providing targeted HIV-prevention interventions for prison inmates, institutional and access barriers have impeded the development and evaluation of such programmes [15].

HIV prevalence in the prisons is usually higher than that in the population at large. It could be 5, 6, or even as much as 10 times higher than the values obtained in the general population [15–17]. A rapid assessment on HIV/AIDS in Nigeria prisons revealed a prevalence rate of 8.7% compared to the national figure of 4.6%. Some other countries in Africa even have higher prevalence with Cote d'Ivoire (27.5%), Zambia (26.7%), and South Africa (15%) [17].

Even though inmates may know that HIV/AIDS could be prevented with the use of condoms, it may not be readily available or affordable [18]. There are considerable proportions of receptive naïve inmates who stand the risk of being infected due to their level of ignorance about HIV/AIDS. Another study stated that there were gaps, misconceptions, and high risk behaviors among prisoners [12]. In a study in Lagos, Nigeria, it was found out that despite the fact that many of these prison inmates knew the correct modes of transmission, many still indulged in high risk behaviors for AIDS transmission [19]. Some studies reported that there are unsafe injecting practices among injecting drug users and the use of nonsterile needles and other cutting instruments is high [20]. Some prison inmates who are professional barbers used unsterilized barbing instruments to barb prisoners because they were unaware of the need for sterilizing these instruments [21].

This study aimed to describe the knowledge, attitude, and preventive practices of male inmates with a view to identify the gaps, misconceptions, and the high risk behaviors towards HIV/AIDS among Ogbomoso Prison inmates in South West Nigeria.

## 2. Materials and Methods

The Ogbomoso Prison is one of the 86 satellite prisons in Nigeria. The satellite prisons are set up mainly in areas with courts that are far from the main prisons. They serve the purpose of providing remand centers especially for whose cases are going on in courts within the area. When convicted, long-term prisoners could be moved to appropriate convict prisons to serve their terms [22].

The study was a descriptive cross-sectional survey conducted in December 2013. The total number of inmates in the prison during the study period was 256 comprising 250 males and 6 females. A total of 167 inmates were recruited for the study after calculating the sample size assuming that 50% of inmates had correct knowledge about HIV in prison using the formula 4 pq/L^2^ and 10% degree precision at 95% confidence interval [23]. Respondents were chosen using simple random sampling method until desired sample size was obtained. None of the females consented to participate in the study. The Survey Select Procedure was used to randomly sample 167 respondents of the 250 males using the full listing of inmates available with prison authority at the time of survey. None of the selected male respondents declined to participate in the study.

Approval for the study was sought from the ethical review committee of Ladoke Akintola University of Technology (LAUTECH) Teaching Hospital (LTH), Ogbomoso, Nigeria. Written permission to interview the inmates was obtained from the prison authorities before the interview. Written informed consent was also obtained from the inmates by signing of the consent forms after the contents of the form had been clearly explained to them. They were also told that the study was voluntary and that individuals who agreed to participate will be allowed to withdraw from the study at any stage of the research.

The instrument for data collection was a pretested structured interviewer-administered questionnaire. A pretest of the instruments was carried out with 17 inmates (10% of the calculated sample size) in Ilorin Prison (about 50 Km from Ogbomoso) with similar sociodemographic characteristics as those of respondents. The questionnaire was adopted from knowledge, attitudes, beliefs, and practices survey of the WHO HIV/AIDS programme and previous literatures [2, 19, 23]. The questionnaire consisted of 39 questions in 4 broad four sections. The first section collected information on sociodemographic profile of the respondents (6 items); the second section obtained information about HIV/AIDS related knowledge (10 items); statements about individual's attitude covering sociocultural issues (7 items); and 16 items about the individual's practices concerning HIV/AIDS. In order to militate against social desirability bias, the indirect questioning was employed where socially desirable response was of special concern especially on sexual behaviors. The questionnaire was written in English but was translated into the three major languages in Nigeria, that is, Yoruba, Hausa, and Igbo languages, to enable the respondents to understand the questions clearly.

The questionnaires were administered by trained research assistants in private rooms made available by the prison authority. The research assistant received one-day training on how to use the questionnaire. The field workers included a resident doctor and undergraduate medical students of the Department of Community Medicine of LTH, Ogbomoso, Nigeria. The researcher spent about two hours with the prison inmates to explain to them the nature of the study. The research assistants helped to fill in the responses of the inmates. Strict confidentiality was maintained.

Evaluation of knowledge of respondents about HIV/AIDS was assessed based on scoring of ten [10] questions that were asked in the questionnaire; a score of one is administered for every right answer while zero is allocated to every wrong answer. The overall mean score obtained was 10 and respondents who scored 10 and above were adjudged to have good knowledge while respondents that scored below ten were said to have poor knowledge.

Data collected were checked for completeness before they were entered into the computer. The data were analyzed using Statistical Package for Social Sciences (SPSS) version 17. Descriptive statistics were applied to determine frequency of relevant variables in the study while Fisher's exact test using a Monte Carlo approach was used to test associations between sociodemographic characteristics and knowledge of the respondents.

The study was limited by being done in only one prison due to logistics and financial constraints; however the findings in this work are expected to give an insight into what prevails in other prisons in Nigeria. Equally, lack of consent by the females' prisoners prevented gender comparison.

## 3. Results

**Figure 1:**
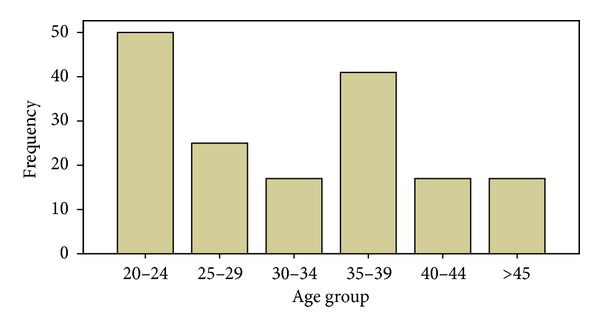
Age groups of inmates in the Ogbomoso Prison, 2013.

The age range of the respondents was 20–59 years (mean = 30.99 ± 11.41). Fifty (29.9%) were in the age group 20–24 years. All the respondents were males. Only 53 (31.7%) had secondary education, 83 (50.3%) were married, and about 67 (40.1%) were drivers before incarceration (Table 1). The highest age groups were found among the age group 20–24 (29.9%) and 35–39 (24.6%) respectively (Figure 1).

All the inmates did not know the meaning of HIV. About 158 (94.6%) of the inmates were aware of HIV/AIDS. Among these, 118 (70.7%) knew that HIV is a virus and 68 (40.7%) knew it is mainly transmitted through unprotected sexual intercourse. About 133 (80%) knew that HIV is transmissible through other modes citing at least one mode of transmission: 139 (83.2%) through infected surgical needles and 75% by using unsterilized sharps such as clippers and blades and infected mother to a child during pregnancy 34 (20.4%). On the risk of HIV infection only 53 (31.7%) believed that the risk of HIV could be reduced by having one faithful partner. About 119 (71.3%) of the respondents believed that a condom protect from both pregnancy and HIV infection (Table 2).

Only 66 (39.5%) of the respondents will offer support and feel sorry for an HIV infected friend, but 115 (68.9%) will avoid an HIV infected friend. About 40 (24%) believed a condom spoil sexual pleasure (Table 3).

Ninety-two (51.1%) believed that HIV infection exists in the prisons. Only 72 (43.1%) believed they are at risk of HIV infection, but most 141 (84.4%) of the respondents are willing to have HIV testing. About 105 (62.9%) of the respondents have had sexual relations; 58 (34.7%) had sexual relation with one regular partner while 44 (26.3%) had sexual relations with more than one partner in the past. As shown in Table 4, about 53 (34.1%) confirmed that the risk of HIV infection can be reduced by having one regular partner. Homosexuality, 124 (74.3%), was recognized as the commonest sexual practice in the prison by respondents. Others include masturbation 34 (20.4%) and intravenous drug injection 9 (5.4%).

Table 5 shows a cross tabulation of the socio-demographic characteristics of respondents with their knowledge about HIV/AIDS and its preventive practices.

## 4. Discussion

The 20–24 years age group represents the largest age group similar to previous studies conducted in South West Nigeria [11, 12] (Figure 1). The religion of the prison inmates does not mirror the general South West population with 80% of the inmates being Christians. Religious beliefs of individuals do have significant bearings on the knowledge and attitudes and beliefs that affect the transmission of HIV/AIDS in both positive and negative ways; for example, the Catholic Christians frown at the use of condoms [13].

Most of the respondents, 158 (94.6%), are aware of HIV/AIDS, with family and friends, 50 (30%), being their main source of information followed by mass media, 42 (25%). The print media, healthcare workers, and the prison officials still have a major role to play in the dissemination of information regarding HIV/AIDS. More programmes concerning HIV/AIDS should be discussed on radio and television frequently since they are major sources of information about health issues in Nigeria.

One hundred and thirty-three (80%) had knowledge that HIV is transmissible. Many of them also know the possible routes of transmission of the virus and identify the sexual route as the commonest route of transmission. Their knowledge is however shallow in some aspects as some believe that hugging (15%), sharing a meal with infected person (25%), mosquito bites (25%), kissing (30%) and witchcraft (20%) were routes of transmission.

About 70% also believed that AIDS can be cured. The more people believe that HIV/AIDS could be cured, the less likely they are to practice safe sex or abstain from risky behavior that increases transmission of the infection [13]. The nonavailability of cure for HIV/AIDS creates fear in the minds of people and motivates them to protect themselves against the disease. Some of the information available to some of the inmates about HIV/AIDS has been incorrect. The primary goal of HIV/AIDS education within the prison systems is to prevent the transmission of HIV as prisoners are potential vector for increased transmission within prison and eventually in the society after their release.

About 65% of the respondents believed that people living with HIV/AIDS (PLWHA) should be avoided. This sort of attitude will enhance stigmatization and discrimination against PLWHA. This will further reduce the rate of voluntary testing for the HIV and militate against self-reporting of status, thereby promoting the spread of HIV infection. About 85% of the respondents would like to be tested for HIV. Voluntary testing and counseling is crucial to the prevention of HIV/AIDS and it is crucial among high risk groups.

Homosexuality is a common sexual practice among the respondents as 75% claimed that it is one of the risky behaviors being practiced in this particular prison although only 5.4% of respondents admitted partaking in any of the risky behaviors including homosexuality. The 5.4% of the respondents that admitted partaking in any of the risky behaviors (including homosexuality) are likely to be severe underestimates considering percentage that claimed that homosexuality is one of the common sexual behaviors practiced in this prison. The practice of homosexuality is a criminal offence in Nigeria unlike many Western countries where it is legal; it carries an additional 14-year jail term in Nigeria when an inmate is convicted. In Nigeria prisons, same sex practices are made possible because inmates of the same sex sleep together in the same cell due to overcrowding which militate against the HIV/AIDS and tuberculosis prevention campaign. Previous studies reported more of such sexual activities in prisons [20, 24, 25]. Prison's officials had acknowledged that homosexuality accounts for over 90% of HIV/AIDS transmission in Nigeria prisons [26]. Previous studies reported that very few of the inmates knew that HIV/AIDS could be contacted through homosexual intercourse [1, 10, 18]. There is the risk of higher rate of HIV transmission in homosexuals compared with heterosexuals, and prisoners engaged in homosexuality are capable of transmitting HIV infection more than those who are not.

## 5. Conclusion and Recommendation

The knowledge about HIV/AIDS among inmates was high, but misconceptions about HIV/AIDS are still rife among the prisoners and educational programs would be required to correct this. Most of the inmates still display negative attitudes that are likely to encourage stigmatization and discrimination against the PLWHA. This will militate against voluntary counseling and testing as fear of isolation will prevent individuals from being tested. Efforts should be made by Nigeria Prison Service to comply with United Nations Committee on crime prevention and control that recommended that each prisoner should be made to occupy by night a cell or room by himself [27]. The sharing of sharp shaving instruments like razor blades should be discouraged in the prison to prevent the spread of HIV among inmates if an instrument got contaminated with blood infected with HIV.

## Figures and Tables

**Table 1:** Sociodemographic characteristics of respondents.

Variables	Frequency	%
Age group (years)		
20–24	50	29.9
25–29	25	15.0
30–34	17	10.2
35–39	41	24.6
40–44	17	10.2
>45	17	10.2
Marital status		
Single	83	49.7
Married	84	50.3
Educational status		
None	8	4.8
Primary school	50	29.9
Completed secondary school	53	31.7
Secondary school dropout	48	28.7
Other	8	4.8
Religion		
Islam	25	15.0
Christianity	134	80.2
Traditional	8	4.8
Ethnicity		
Yoruba	107	64.1
Hausa/Fulani	26	15.6
Igbo	16	9.6
Others	18	10.8

**Table 2:** Knowledge of HIV/AIDS by respondents.

Questions	Frequency of “Yes answers”	%
Have you ever received information on HIV/AIDS?	158	94.6
What do you think causes HIV/AIDS?		
Virus	118	70.7
Punishment from God	41	25.0
Is HIV transmissible?	133	80
Can HIV/AIDS be cured?	60	35.9
Can healthy-looking person have HIV?	159	90.2
Will a condom protect from pregnancy and HIV?	119	71.3
Will a condom protect from pregnancy but not HIV?	53	31.7
∗Mode of transmission of HIV mode		
Hugging or shaking of hands	74	44.3
Having sexual intercourse without a condom	68	40.7
Sharing a meal with an infected person	118	70.7
Mosquito bites	98	58.7
A mother infected with HIV to unborn baby	34	20.4
Kissing someone infected with HIV	120	71.9
Do you know that you can be infected with HIV/AIDS during injection?	165	90.8
Spiritual/witchcraft	35	21.0
Using surgical needles containing infected blood	139	83.2
Using of unsterilized sharps (clippers and blades)	36	21.6
Risk of HIV infection can be reduced by having one faithful partner	53	31.7

∗Multiple answers.

**Table 3:** Attitudes towards HIV prevention by respondents.

Questions	Frequency of “Yes answers”	%
Do you know anyone infected with HIV?	143	85.6
Would you offer support to an HIV infected friend?	66	39.5
Would you avoid an HIV infected friend?	115	68.9
Would you use the same WC with HIV infected person?	61	70
Will a condom spoil sexual pleasure?	40	24.0

**Table 4:** HIV prevention practices by respondents.

Questions	Frequency of “Yes answers”	%
Have you ever used a condom?	83	49.7
In the past year have you had sexual relations?	105	62.9
Did you use a condom last time you had sex?	47	28.1
In the past year have you had sexual relation with one regular partner?	58	34.7
In the past year have you had sexual relations with more than one partner?	44	26.3
Do you feel you are at risk of an HIV/AIDS infection?	72	43.1
Have you ever had an HIV test?	26	15.5
Would you like to have an HIV test?	141	84.4
Do you know where to have HIV test?	115	68.9
Do you believe HIV/AIDS exist in prison?	92	55.1
Which of these risky behaviors exist in this prison?		
Homosexuality	124	74.3
Masturbation	34	20.4
Intravenous drug injection	9	5.4
Do you know inmate who has used hard drugs?	113	67.3
Do you know of the inmates who practice anal sex in the prison?	25	15.0
Do you partake in any of the risky behaviors mentioned above?	9	5.4

**Table 5:** Sociodemographic characteristics of respondents and knowledge about HIV/AIDS.

Variables	Good knowledge	Poor knowledge
Age groups (years)		
20–24	50	0
25–29	16	9
30–34	17	0
35–39	41	0
40–44	17	0
>45	17	0
Marital status		
Single	83	0
Married	75	9
Educational status		
None	8	0
Primary	50	0
Completed secondary school	44	9
Secondary school dropout	48	0
Others	8	0
Occupation before incarceration		
Driving	67	0
Schooling	25	0
Mechanic	8	0
Farming	8	0
Carpentry	8	0
Others	42	9
